# Expert panel opinion on the optimal educational pathway for diabetes educators for training people with type 1 diabetes on the MiniMed™ 780G system: a Delphi consensus

**DOI:** 10.1007/s00592-024-02388-w

**Published:** 2024-10-29

**Authors:** Geraldine Gallen, Alice Rosso, Núria Alonso-Carril, Sima Arbeli, Virginie Bahon, Vanessa Brown, Kerstin Endlich, Francesca Gulotta, Audrey Hansart, Amy Jolley, Rea Jussila, Anna Stefanowicz-Bielska, Paola Cardano

**Affiliations:** 1 Diabetes Department, Kings College Hospital, London, UK; 2 Department of Public Health Sciences and Paediatrics, Regina Margherita Children’s Hospital, Turin, Italy; 3 Endocrinology and Nutrition, University Hospital Mútua Terrassa, Terrassa, Spain; 4 Diabetes Department, Maccabi Health Fund, Tel Aviv, Israel; 5 Independent Diabetes Nurse, Lausanne, Switzerland; 6 Centre for Diabetes and Endocrinology, Johannesburg, South Africa; 7 Centre of Dr. Med. Ralf Kolassa, Bergheim, Germany; 8 Diabetes EMEA, Medtronic Interantional Trading Sarl, Tolochenaz, Switzerland; 9 Diabetesteam Kinderen & Diabeteskliniek, UZ Brussel, Brussels, Belgium; 10 Salford Integrated Diabetes Team, Salford Care Organisation, Part of the NCA, Salford, England; 11 Diabetes Center, Paediatric Outpatient Clinic, HUS Jorvi Hospital, Espoo, Finland; 12 Division of Internal and Pediatric Nursing, Institute of Nursing and Midwifery, Faculty of Health Sciences with the Institute of Maritime and Tropical Medicine, Medical University of Gdansk, Gdansk, Poland; 13 Department of Paediatrics, Diabetology and Endocrinology, University Clinical Center in Gdansk, Gdansk, Poland; 14 Medtronic Clinical & Regulatory Solutions - Study & Scientific Solutions, Roma, Italy

**Keywords:** Diabetes educators, Diabetes nurses, Dieticians, Educational pathway, MiniMed™ 780G system

## Abstract

**Introduction:**

The MiniMed™ 780G system is an advanced insulin pump system incorporating an AHCL (Advanced Hybrid Close Loop) algorithm that automatically adjusts insulin delivery based on glucose levels. All guidelines recommend the use of Automated Insulin Delivery therapy for people with type 1 diabetes (PWT1D) and they highlight that a specifically trained and expert team should provide training on HCL systems for PWT1D, but none of the publications detail the curriculum profile that diabetes educators should have. This paper aims to establish a consensus on the optimal educational pathway for diabetes educators on the MiniMed™ 780G system.

**Methods:**

An Expert Panel (EP) of 11 key opinion educators in diabetes technology in the EMEA area was assembled. Using the Delphi method, a consensus questionnaire based on the literature research was created, discussed and validated by the EP members. An agreement level of ≥ 75% was considered a strong consensus.

**Results:**

EP members had on average 16.3 years of clinical experience and followed at least 50 PWT1D using the MiniMed™ 780G system. All EP members agreed that a structured educational pathway to train diabetes educators in the use of the MiniMed™ 780G system is needed. 100% of the EP members agreed that the pathway should include a mentorship programme and in-field training; 90% agreed on using face-to-face training with the support of e-learning modules. The EP members believed that minimum competency standards for diabetes educators around the principles of diabetes care and education are needed.

**Conclusion:**

The educational pathway created by the EP showed that skills are needed at an advanced level and that mentorship in developing these skills is critical. This pathway is vital for supporting the implementation of diabetes technology into everyday life and can remove barriers and increase access to PWT1D.

## Introduction

The MiniMed™ 780G system is an advanced insulin pump system, designed for diabetes management. It incorporates an Advanced Hybrid Closed Loop (AHCL) algorithm that automatically adjusts insulin delivery based on glucose levels. One recent randomized trial demonstrated significant improvements in HbA1c and average glucose levels for people with type 1 diabetes (PWT1D) with HbA1c levels greater than 8.0% (64 mmol/mL) compared to a group using multiple daily insulin injections plus CGM [1]. A study with patients naïve to technology showed that the MiniMed™ 780G system lowered HbA1c levels and significantly increased time in range (TIR) while reducing time below range (TBR). The same trial has also demonstrated positive effects on patients’ psychological well-being [2, 3]. Furthermore, a single centre experience randomized trial demonstrated that also using a simplified meal announcement protocol, international glycaemic targets could be achieved [4]. 

Advances in HCL technology have revolutionised the management and treatment of type 1 diabetes becoming the therapy of choice for most PWT1D. All published guidelines recommend that training in the use of HCL systems to PWT1D should be provided by a multidisciplinary team, specifically trained and experienced in the use of this technology. The team should receive ongoing, comprehensive, and structured training, with continuous updates [5–10]. However, none of the publications detail the curriculum profile that diabetes educators should have to provide education and training in the use of AHCL technology like MiniMed™ 780G system. As diabetes educator we refer to diabetes specialist nurses/dietitians.^7^

Continued education, support and mentoring of diabetes educators are essential to improve both health outcomes and quality of life for people with diabetes [5]. It is important to establish minimum competence standards for diabetes educators, principles of diabetes care and education, and checklists to assess their knowledge and skills in the field of the AHCL systems.

Properly conducted therapeutic education helps to fully involve PWT1D in both self-control and improvement of their treatment outcomes.

This paper aimed to establish a consensus on the educational pathway that diabetes educators should follow to become competent in the care and education of people with diabetes, families, and caregivers who use the MiniMed™ 780G systems. The Delphi method [11, 12] was used to achieve consensus among a panel of 11 diabetes educators, experts in diabetes technologies, from Europe, the Middle East, and Africa (EMEA).

The resulting educational pathway is intended to guide healthcare professionals (HCPs) involved in AHCL education.

## Methods

### Expert panel

The consensus topic for the focus of this paper was the optimal educational pathway for diabetes educators willing to onboard and follow PWT1D on the MiniMed™ 780G system. The expert panel (EP) members, who wrote this consensus, were identified as being key opinion leaders (KOLs) in the field of diabetes technology implementation across EMEA. 11 EP members from 10 countries volunteered to join the panel and provide their expert opinions. They were selected by Medtronic according to their experience with onboarding PWT1D to the MiniMed™ 780G system and already following at least 50 users.

### ​ Delphi method

The Delphi method [11, 12], which is a systematic process of forecasting using the collective opinion of panel members in areas in which evidence is scarce, was used to reach a consensus. Consensus is reached by one or more consecutive survey questionnaires and discussions between the EP members until they reach an agreement on all the topics.

The EP members met virtually to discuss the unmet needs about the topic and produced statements, which were in serious need of clarification and debate. Lead panel member, Geraldine Gallen, Type 1 Service Lead at Kings College Hospital - London, developed a questionnaire with 7 consensus questions based on literature research results. The draft questionnaire was distributed in November 2023 for review and validation. The EP members met online to agree on the final survey on 27th November 2023. During this meeting, they decided to modify the formulation of some questions to clarify their meaning and add more questions to gather more detail on certain topics. The EP members were requested to complete the survey by the 15th of December 2023. All 11 EP members completed the survey.

The consensus was composed of 7 questions to evaluate the level of agreement or disagreement with each statement and a 5-point Likert scale, scored as follows: (1) strongly disagree; (2) disagree; (3) neutral; (4) agree; (5) strongly agree, was used. A strong consensus on a specific question was reached if at least 75% of EP members strongly agree or agree on that question. (Table 1). For almost each consensus question, a supplementary question was included to qualify the EP’s experience in diabetes, technology and education and the current barriers for diabetes educators in their country (Table 1).

**Table 1:** Survey questions

Consensus questions	Qualifying and supplementary questions
1. Do you agree that there is a need for a comprehensive and structured educational pathway for diabetes educators who train and educate T1D patients on MiniMed™ 780G system technology?	**Current barriers**: Time constraints Financial constraints Lack of exposure to education opportunities Lack of team support Lack of type 1 experience Other
2. Do you agree that a mentorship programme should be part “of “The Pathway”	No supplementary question
3. Do you agree that F2F trainings are needed to be part of “The Pathway”	**Specification**: Number Duration
4. Do you agree that e-learning modules are needed to be part of “The Pathway2	**Time**: Pre-training Anytime during the learning process Recap Material Continue learning material
5. Do you agree that in-field training (observing and conduct supervised training with a patient) is needed to be part of “The Pathway”	**Specification**: Number Training Materials
6. Do you agree that training on therapy clinical evidence should be a part of “The Pathway”?	No supplementary question
7. Do you agree that the completion of “The Pathway” has to be certified by a competent scientific body	**Specification**: Level of Certification Suggestion of Certification Body

Two supplementary questions were included: (1) the minimum level of knowledge of the MiniMed™ 780G system and (2) an open question for describing the ideal educational pathway.

A virtual meeting was held at the end of January 2024 to present and discuss the results of the final survey.

### ​ Statistical analysis

To summarise the survey results, data are expressed as mean *±* standard deviation for continuous data or as fractions and percentages for categorical data. Excel Office 365 (Microsoft, Redmond, Washington) was used to perform analyses.

## Results

### Literature research

Few studies are available on which educational pathway diabetes educators need to follow to become experts.

A qualitative ethnographic study from James (2016) on Australian Diabetes Educator’s perceived experiences with diabetes technology showed that nurses found the use of technology burdensome and they expressed needing help in supporting patients with using technology. Lack of skilled staff, time constraints and keeping up to date with advances in technology were seen as barriers. The findings suggest that to maximize technology adoption, diabetes educators need support in attaining and retaining the necessary skills [13]. 

The American Association of Diabetes Educators published in 2023 a piece on the evolving role of the diabetes educator in population health. It was highlighted that there is increasing use of technology in diabetes and that diabetes educators need to be well-versed in using technological devices and know how to interpret data for people with diabetes as well as other health professionals [5]. 

According to the study by Patil et al. (2022), it is crucial for quality diabetes care to integrate diabetes technology knowledge for healthcare professionals, who in turn enables patients to improve clinical outcomes and reduce diabetes distress by getting the competency to include diabetes technology successfully into self-care. The field of medical and diabetes technology is rapidly developing, which makes it crucial to ensure the knowledge and competency of healthcare professionals stays up-to-date and prioritized. This includes all members of the healthcare team, as well as support staff and the patients [14]. 

### EP members characteristics

The characteristics and current diabetes technology practices of the 11 diabetes educators (8 nurses, 2 dieticians and 1 diabetes educator) who participated in the expert panel were as follows:

- Average clinical experience is 16.3 *±* 9.6 years.
- 91% (10 out of 11) EP members have post-graduate qualifications in diabetes management.
- 79.7% of PWT1D are onboarded on pump therapy face-to-face.
- 13.8% of PWT1D are onboarded on pump therapy remotely.
- 6.5% of PWT1D are onboarded on pump therapy using an hybrid approach (face-to-face and remote).
- 69.4% of pump trainings are managed individually.
- 30.6% of pump trainings are managed in groups.

**Fig. 1:**
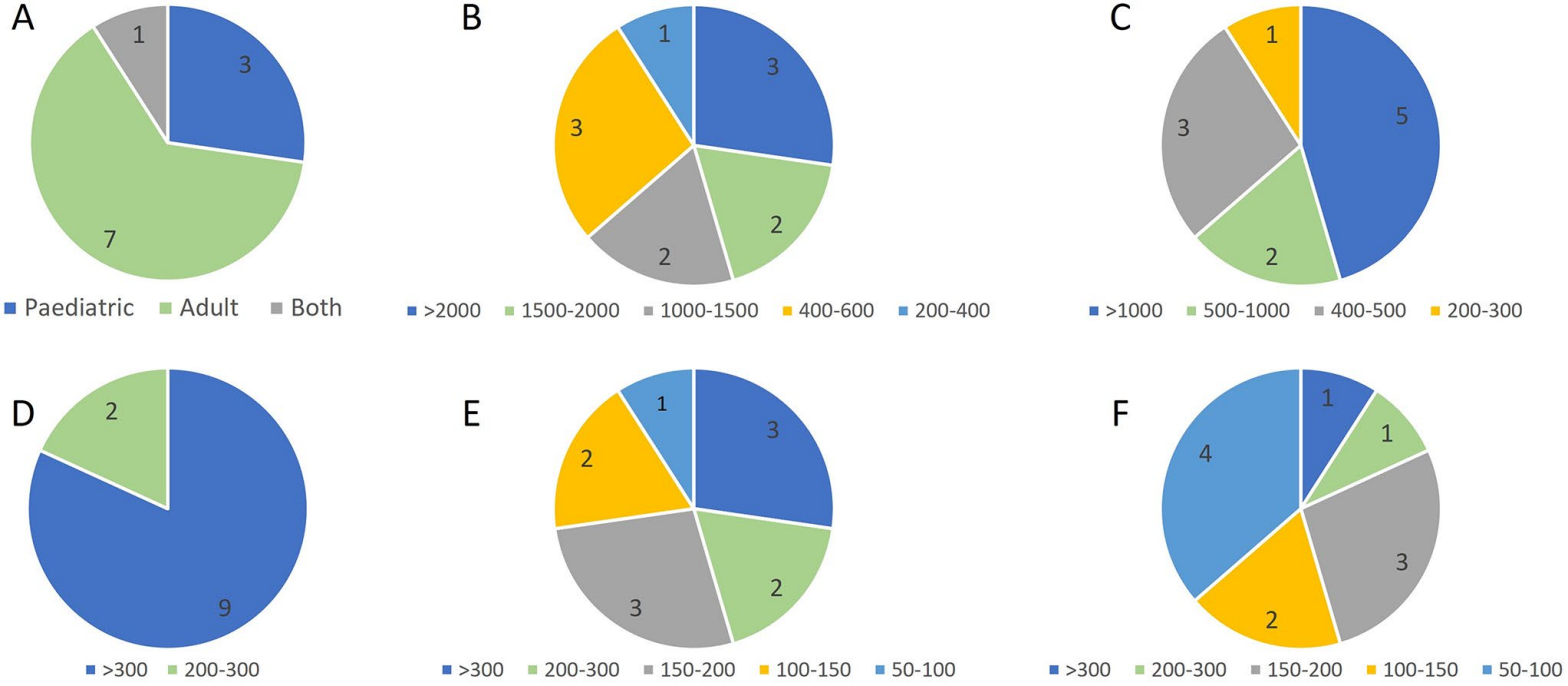
EP members’ experience profile in following people with diabetes according to A**A**: number of people with diabetes by age group, **B**: number of people with diabetes attending the diabetology clinic, **C**: number of PWT1D, **D**: number of PWT1D who were using technology, **E**: number of PWT1D who were using HCL technology and **F**: number of PWT1D who were using MiniMed™ 780G system. EP, expert panel; PWT1D, people with type 1 diabetes; HCL, Hybrid closed loop

At the time of the consensus, most EP members (7) were involved in following adult people with diabetes, only 3 members followed paediatric people, and 1 was involved in both adult and paediatric diabetes care (Fig. 1).

All EP members were involved in caring for large numbers of people with diabetes as well as PWT1D (Fig. 1).

Most EP members followed more than 300 PWT1D who were using diabetes technology and more than 150 using an AHCL system (Fig. 1).

As requested by the criteria to be part of this EP, all the members had vast experience in following PWT1D using the MiniMed™ 780G system (Fig. 1).

### Structure of the educational pathway

According to the Delphi method, the threshold for a strong consensus was set to 75% of EP members who strongly agree or agree on a specific question. Strong consensus was reached for the majority of questions relating to the structure of the educational pathway.

**Fig. 2:**
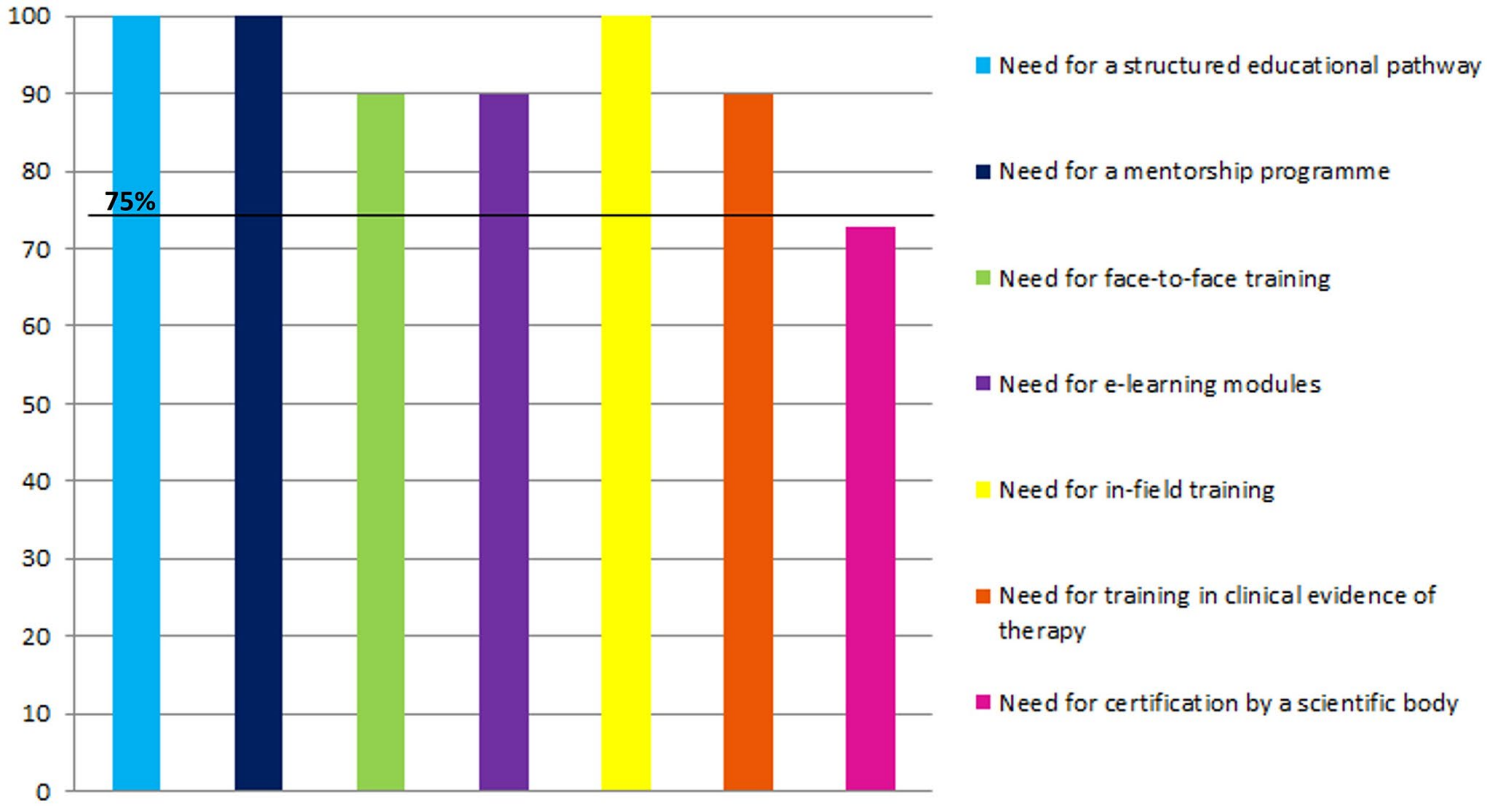
Consensus questions results. The black line displays the threshold for a strong consensus (75% of EP members who strongly agree or agree on a specific question)

All 11 EP members agreed that a structured educational pathway is needed to train diabetes educators in the use of the MiniMed™ 780G insulin pump system, including PWT1D on the system and providing ongoing support for these users (Fig. 2).

100% of EP members agreed that a mentorship programme is needed for ongoing support as well as in-field training, i.e. training during the educator’s first few patient onboarding sessions (Fig. 2).

90% of EP members agreed that training needs to be face-to-face with the support of e-learning modules and that education on clinical evidence on the therapy must be part of the pathway (Fig. 2).

After the survey completion, a strong consensus was not reached on whether the educational pathway needs to be certified by a scientific regulatory body. During the online meeting to review the survey answers, EP members agreed that there should be certification, but it is premature to define which scientific body could be used in the different countries or at the international level.

### Current barriers to accessing the necessary education on diabetes technology

100% of EP members agreed that currently there are time constraints to obtaining the necessary education on diabetes technology, 82% agreed that there is a lack of education opportunities and 73% agreed that there are financial constraints. There was low consensus regarding team support (45%) and lack of experience with treating PWT1D (36%). Other barriers listed by EP members were: difficulty in getting permission from their employer to participate in training, training in pump therapy not currently being part of their job description, and being required to work in many areas of diabetes management including type 2 diabetes and gestational diabetes which reduced focus on specialized type 1 diabetes treatments, lack of real-life experience with the MiniMed™ 780G system.

### Need for a mentorship programme

There was 100% agreement that a mentorship programme should be included as part of the educational pathway. The EP members suggested that the mentor role should be performed by a diabetes educator who has a high level of expertise in the management of PWT1D and who is competent in the use and management of AHCL therapy, such as the MiniMed™ 780G system. They should be involved in an operational diabetes technology service and provide a source of expertise as needed during the educational pathway. They should be responsible for giving structured feedback and identifying areas for further training. The mentorship programme should include clear goals, regular check-ins, tailored guidance, and opportunities for hands-on experience. It could be offered face-to-face where possible or in an online virtual format. There should be room for case discussion.

### Need for face-to-face training

90% of EP members agreed that face-to-face training is needed. Considering the optimal number of sessions, 18% of the EP members agreed on 1–2 sessions, 55% on 2–3 sessions, 18% on 4 or more sessions and one participant suggested it should be guided by self-assessment or by assessment from a mentor. In terms of the duration of each face-to-face session, 18.5% agreed on the sessions lasting less than 2 h, the majority (46%) on 2 h and 36% on more than 3 h. During the online meeting to review the survey answers, there was 100% agreement on 2–3 face-to-face sessions of 2 h each.

### Need for e-learning modules

There was a 90% consensus that e-learning modules should be included in the pathway. Considering the different steps of the pathway, 8 out of 11 EP members said it should occur at any time during the pathway, 3 for pre-training, 1 for recapping of material and there was 100% consensus that e-learning modules should be available after certification on a continued basis.

### Need for in-field training

There was a 100% consensus that in-field training is needed as part of the educational pathway. Considering the number of sessions needed, answers ranged between 1 and 5. During the January online meeting, there was a 100% consensus that there should be at least 2 sessions and the mentor would assess if more were needed. A possible suggested format was to watch one full onboarding session, co-train one session, and then run one session with supervision.

### Type of training material to be provided by the pathway

As part of the survey, there was a question on which material can be provided during the educational pathway, and all 11 EP members suggested PowerPoint presentations and one-page summaries, 10 agreed on using guides, videos, and scientific articles, 4 on using podcasts and 3 on books. There were two further suggestions for using a virtual demo pump and pump therapy International Guidelines as part of the educational pathway.

### Level of knowledge per topic needed to be acquired by the pathway

As part of the consensus survey, the 11 EP members answered a questionnaire related to the minimum knowledge needed to be acquired during “The Pathway” grouped by topics and commented through an open question if there were any other topics that needed to be added to the minimum knowledge list.

The levels of knowledge were divided into three levels: Low knowledge, Medium knowledge and High knowledge and all EP members marked their opinion of the level of knowledge needed.

The level of knowledge per topic and the EP members answers are listed in Table 2.

**Table 2:** Level of knowledge per topic

**MiniMed™ 780G pump features**		**Low knowledge**	**Medium knowledge**	**High knowledge**
How to transition from MDI to pump therapy (initial settings)		0	0	11
How an insulin pump works		1	0	10
Infusion set issues/sites		0	0	11
Initial pump settings		0	1	10
How the Bolus Calculator works		1	0	10
Alerts & Alarms		0	1	10
Treating lows and highs on the pump		0	0	11
Insulin pump troubleshooting		0	1	10
**MiniMed™ 780G CGM features**	**Low knowledge**		**Medium knowledge**	**High knowledge**
How a CGM works	1		0	10
Why is automated insulin delivery needed?	0		1	10
Manual mode features	0		0	11
How SmartGuard™ feauture works	0		0	11
SmartGuard™ settings	0		0	11
Temporary target	0		0	11
How to switch back to MDI	0		0	11
CGM troubleshooting	0		3	8
**Meal handling**	**Low knowledge**		**Medium knowledge**	**High knowledge**
Carb Counting	1		3	7
High-fat/Protein foods	1		4	6
How to use a bolus calculator	0		0	11
**CareLink**^**TM**^ software	**Low knowledge**		**Medium knowledge**	**High knowledge**
How to create a CareLink™ Personal account	0		5	6
CareLink™ Data interpretation and pump setting adjustment	0		0	11
**Special considerations**	**Low knowledge**		**Medium knowledge**	**High knowledge**
How to manage exercise	0		2	9
How to manage sick days	0		0	11
How to manage alcohol	0		2	9
How to manage gastroparesis	0		4	7
How to manage skin integrity	0		2	9
How to manage travel	0		1	10

A very high consensus between EP members was reached for most of the topics (Table 2).

The EP also raised additional topics to be added to the table: prolonged fasting, pregnancy and diagnostic examinations. These matters could be considered in the future.

### Survey results of the open question regarding the ideal pathway

In the last survey question, EP members provided their ideas on how an optimal educational pathway would look like. The results have been collected and categorised in Table 3.

**Table 3:** EP members’ proposal for the “Optimal educational pathway”

The pathway structure	• Easily accessible: accessible to all interested parties and free. • Available in virtual/digital and F2F formats and easy to use. • Logical in structure and contains all the necessary topics for training with the opportunity to intensify training knowledge, including a checklist. • Designed in a way that diabetes educators or pump therapy trainers can use it to achieve a technical briefing, regardless of their level of knowledge and experience. • Equipped with a final test to evaluate the knowledge and skills acquired. • Continued access to online material to recap learning and new modules added as new technology is launched.
Tools to help to successfully complete the pathway	• Opportunity to wear pump and sensor for a week to practice, • Assign a mentor/supervisor who can provide feedback and support post-training. • First MiniMed™ 780G system onboarding with supervision. • Combination of online and in person-training simultaneously. • Access to a virtual pump application to practice what is taught in the face-to-face session.
Post-certification	• Continued access to online material to recap learning and new modules added as new technology is launched.

## Discussion

The primary aim of this consensus was to develop an optimal educational pathway for diabetes educators to follow when learning how best to onboard and follow-up PWT1D using the MiniMed™ 780G system.

Examining the lack of publications and guidance in this area, an expert consensus using a systematic methodology was considered to be a useful tool in clinical practice. As far as we are aware, this is the first paper that delivers a detailed pathway with competencies, including levels of knowledge needed in specific areas around a device. As all EP members had extensive experience with the MiniMed™ 780G system in particular, they decided to focus the consensus on the use of this device, but the EP members believe that most of the recommendations could be applied to other AID systems.

To achieve consensus EP members decided to use the Delphi method, a flexible and adaptable but systematic tool to gather and analyse the needed data about a specific topic [15]. 

The selection of educators to be experts was crucial for this project as it determined the validity and likeliness of leading to a consensus and an educational pathway that can be used across EMEA. The consensus results were a testament to their experience with the MiniMed™ 780G system. Shang [16] in a recent review on the use of Delphi in health science defined an expert as someone who can provide high-quality responses in a particular area, and it highlighted that the number of panellists can range from as few as 4 to several thousand. Most commonly, Delphi consensus is managed by panels composed of 8 to 20 members.

A strength of this project was the high level of consensus and stability that was achieved for the majority of questions relating to the need for a structured educational pathway based on competencies, highlighting the current gap in training in all EMEA countries.

All EP members agreed that a structured educational pathway for initiating and supporting PWT1D to use the MiniMed™ 780G insulin pump system was needed and that a mentorship programme for ongoing support as well as in-field training should be provided.

This was also the conclusion from an American group [14] that examined the integration of diabetes technology into the patient pathway; they recommended a pathway with professional competencies as a necessity as this would serve as a roadmap to the knowledge and skills required by educators but would also help define a standard of care and identified the scope of practice. An Australian paper [13] looking at patient barriers to technology concurred adding that educators need support to attain and retain the skills required to deliver these essential components of care but would achieve those through more technology exposure to align service delivery and greater consistency of patient experience.

Both groups highlighted the need for mentorship programmes for educators but did not elaborate on what that relationship would look like. The EP members defined this role as an experienced diabetes educator who is competent in the advanced use of the MiniMed^™^ 780G system, but they also felt that the mentor should be involved in operational diabetes technology services and would be a source of expertise due to this experience. The mentor should support the educator with training opportunities in their clinic (virtually or in person) and provide a structured programme which includes clear goals, regular visits tailored to educators’ needs, guidance, and opportunities to gain hands-on experience. The significance of mentorship within healthcare training is well established. It presents an opportunity to enhance the educators’ performance and engagement, promote learning opportunities and encourage multidisciplinary collaboration [17]. 

The EP members agreed that diabetes educators should have a clear knowledge of the clinical evidence: understanding how the technology can be involved in all aspects of health, including eligibility, affordability, safety, patient acceptability, ethical implications, and clinical effectiveness in comparison with traditional or other treatments is critical. The EP believed that diabetes educators should be required to regularly update their practical training and theoretical knowledge of technology-related guidelines.

The type of training material that should be used within the pathway was varied across all EP members with a mix of PowerPoint presentations, one-page summaries, user guides, videos, and scientific articles.

Bae et al. [18] found that when interviewing nurse practitioners there was no ideal training material or method and was dependent on the training purpose and preferences of the educators. We know, however, that providing a variety of educational materials also comes at a cost to the healthcare provider. Maloney et al. [19] looked at the cost-effectiveness of education delivery for HCPs and found that web-based educational materials and programmes are robustly superior in terms of cost-saving when compared to face-to-face interventions. The EP members will need to negotiate a balance within the pathway materials and look for already available resources to ensure that the pathway can be delivered at scale in different countries in different languages.

A strong consensus was not reached on whether the educational pathway needs to be certified by a scientific regulatory body, the EP members did however agree that there should be certification but were unsure how this could be done in the different countries or at the international level and this topic remained as a long-term aim for the EP members and the educational pathway.

HCP educational programmes, supervision and proficiencies are traditionally regulated by bodies which set standards for practice [20]. Clinical pathways are tools used to guide evidence-based healthcare and they aim to translate and standardise clinical guideline recommendations into clinical processes of care [21].

As part of the consensus survey, EP members answered questions related to the minimum knowledge needed to be acquired during the educational pathway grouped by topics. The EP members believed that it was important to establish minimum competency standards for diabetes educators around the principles of diabetes care and education, using checklists to assess the knowledge and skills in the field of the AHCL system. A very high consensus on the level of knowledge of tasks and education was noted on all 27 topics.

In the final section of the questionnaire, participants provided their recommendations regarding the optimal educational pathway for diabetes technology, as outlined in Table 3. This table emphasizes three critical areas: the pathway’s structure, supportive tools for successful completion, and post-certification considerations.

The next steps for the EP members will include the adoption and implementation of the educational pathway into practice as validation of its success. The EP were also aware that as new technology is developed and the competencies are implemented, the EP members will need to conduct ongoing evaluations and revisions to ensure that the diabetes technology pathway continues to meet the needs of diabetes educators, and where possible other HCL systems to avoid multiple pathways.

## Limitations

The EP members, coming from large established technology services and represented both international adult and paediatric populations, are aware that the pathway may not be exactly suitable for smaller diabetes services that are at the early stages of implementing technology into their treatment pathway and adaptation of this would largely depend on the expertise and facilities within those centres and availability of experienced mentors. Where this may be the case a hub and spoke model may need to be adapted.

The pathway focused on providing education on the technical features of MiniMed™ 780G system and the therapy management, but it did not consider other important aspects of the diabetes educators’ education such as communication techniques [22] and relational skills.

The pathway did not highlight who is ultimately responsible for the training and education of diabetes educators, i.e. where the responsibility from industry ends and where responsibility from the clinical team start. The EP members felt that this needed to be a joint approach and that both partners have a role to ensure that evidence-based education is available and kept up to date.

Currently, industry partners are offering face-to-face and online training for all diabetes centers on their devices.

The ultimate responsibility for education should lie with the clinical team to ensure all HCP’s involved in diabetes technology receive the necessary education and that they are upskilled with the clinical knowledge needed to manage PWT1D using technology. Resources are also provided by expert groups such as the Diabetes Technology Network - UK (DTN-UK) or shared in platforms such as the Advanced Technology and Treatments for Diabetes (ATTD) educational portal, both of which have produced and presented best practice guides on the clinical use of AHCL.

## Conclusion

In this manuscript, we identified an educational pathway vital for supporting the implementation of diabetes technology into everyday life and for removing barriers and increase access to PWT1D.
