# Correction: Expert panel opinion on the optimal educational pathway for diabetes educators for training people with type 1 diabetes on the MiniMed™ 780G system: a Delphi consensus

**DOI:** 10.1007/s00592-024-02435-6

**Published:** 2024-12-26

**Authors:** Geraldine Gallen, Alice Rosso, Núria Alonso Carril, Sima Arbeli, Virginie Bahon, Vanessa Brown, Kerstin Endlich, Francesca Gulotta, Audrey Hansart, Amy Jolley, Rea Jussila, Anna Stefanowicz-Bielska, Paola Cardano

**Affiliations:** 1 Diabetes Department, Kings College Hospital, London, UK; 2 Department of Public Health Sciences and Paediatrics, Regina Margherita Children’s Hospital, Turin, Italy; 3 Endocrinology and Nutrition, University Hospital Mútua Terrassa, Terrassa, Spain; 4 Diabetes Department, Maccabi Health Fund, Tel Aviv, Israel; 5 Independent Diabetes Nurse, Lausanne, Switzerland; 6 Centre for Diabetes and Endocrinology, Johannesburg, South Africa; 7 Centre of Dr. Med. Ralf Kolassa, Bergheim, Germany; 8 Diabetes EMEA, Medtronic Interantional Trading Sarl, Bergheim, Germany; 9 Diabetesteam Kinderen & Diabeteskliniek, UZ Brussel, Brussels, Belgium; 10 Salford Integrated Diabetes Team, Salford Care Organisation, Part of the NCA, Salford, England; 11 Diabetes Center, Paediatric Outpatient Clinic, HUS Jorvi Hospital, Espoo, Finland; 12 Division of Internal and Pediatric Nursing, Institute of Nursing and Midwifery, Faculty of Health Sciences with the Institute of Maritime and Tropical Medicine, Medical University of Gdansk, Gdansk, Poland; 13 Department of Paediatrics, Diabetology and Endocrinology, University Clinical Center in Gdansk, Gdansk, Poland; 14 Medtronic Clinical & Regulatory Solutions - Study & Scientific Solutions, Roma, Italy

**Correction: Acta Diabetologica**

10.1007/s00592-024-02388-w.

In the original version of this article, the given and family names of author names were incorrectly structured. The correct given name and family name should be Geraldine Gallen^1^, Alice Rosso^2^, Núria Alonso-Carril ^3^, Sima Arbeli^4^, Virginie Bahon^5^, Vanessa Brown^6^, Kerstin Endlich^7^, Francesca Gulotta^8^, Audrey Hansart^9^, Amy Jolley^10^, Rea Jussila^11^, Anna Stefanowicz-Bielska ^12,13^ and Paola Cardano^14^

The original article has been corrected.

